# Molecular Association Assay Systems for Imaging Protein–Protein Interactions in Mammalian Cells

**DOI:** 10.3390/bios15050299

**Published:** 2025-05-08

**Authors:** Sung-Bae Kim, Tadaomi Furuta, Suresh Thangudu, Arutselvan Natarajan, Ramasamy Paulmurugan

**Affiliations:** 1 Environmental Management Research Institute, National Institute of Advanced Industrial Science and Technology (AIST), 16-1 Onogawa, Tsukuba 305-8569, Japan; 2 School of Life Science and Technology, Institute of Science Tokyo, B-62 4259 Nagatsuta-cho, Midori-ku, Yokohama 226-8501, Japan; furuta@bio.titech.ac.jp; 3 Molecular Imaging Program at Stanford, Bio-X Program, Stanford University School of Medicine, Palo Alto, CA 94304, USA; suresh07@stanford.edu (S.T.); anataraj@stanford.edu (A.N.); paulmur8@stanford.edu (R.P.)

**Keywords:** molecular association assay (MAA), protein–protein interactions (PPIs), *Renilla* luciferase 8.6SG (R86SG), artificial luciferase (ALuc), bioluminescence (BL)

## Abstract

Molecular imaging probes play a pivotal role in assaying molecular events in various physiological systems. In this study, we demonstrate a new genre of bioluminescent probes for imaging protein–protein interactions (PPIs) in mammalian cells, named the molecular association assay (MAA) probe. The MAA probe is designed to be as simple as a full-length marine luciferase fused to a protein of interest with a flexible linker. This simple fusion protein alone surprisingly works by recognizing a specific ligand, interacting with a counterpart protein of the PPI, and developing bioluminescence (BL) in mammalian cells. We made use of an artificial intelligence (AI) tool to simulate the binding modes and working mechanisms. Our AlphaFold-based analysis on the binding mode suggests that the hinge region of the MAA probe is flexible before ligand binding but becomes stiff after ligand binding and protein association. The sensorial properties of representative MAA probes, FRB-ALuc23 and FRB-R86SG, are characterized with respect to the quantitative feature, BL spectrum, and in vivo tumor imaging using xenografted mice. Our AI-based simulation of the working mechanisms reveals that the association of MAA probes with the other proteins works in a way to facilitate the substrate’s access to the active sites of the luciferase (ALuc23 or R86SG). We prove that the concept of MAA is generally applicable to other examples, such as the ALuc16- or R86SG-fused estrogen receptor ligand-binding domain (ER LBD). Considering the versatility of this conceptionally unique and distinctive molecular imaging probe compared to conventional ones, we are expecting the widespread application of these probes as a new imaging repertoire to determine PPIs in living organisms.

## 1. Introduction

Molecular imaging probes have been broadly used as tools for assaying various molecular events in cells and living animals. Historically, the probes have been designed to determine protein–protein interactions (PPIs) and to generate optical signals [1,2].

The common optical signals in the probes are fluorescence (FL) and bioluminescence (BL). Both optical signals have unique merits and demerits as an optical readout in bioassays, and they can compensate for each other in multiplex bioassay systems [3]. The BL commonly used in the probes is produced by the oxidative reaction of a luciferase with its specific substrate luciferin in the presence of molecular oxygen [4]. These bioluminescent probes are generally advantageous with respect to a long linear dynamic range, low background intensity, a high signal-to-background (S/B) ratio, biocompatibility, simplicity in signal development, and versatility in biological systems [5].

To date, several conceptually important bioluminescent probes have been developed to assay molecular events in mammalian cells. The most conventional and conceptually unique one for imaging PPIs may be the “two-hybrid assay system” using a split-transcription factor [6]. This system makes use of an expression vector encoding a specific promoter sequence, which is linked to the coding region that regulates the transcription of the reporter luciferase. A ligand-activated PPI triggers the reconstitution of a transcription factor, which binds to the promoter sequence and activates the expression of the reporter luciferase or fluorescent protein as an optical readout. However, this assay system generally has the following demerits: (i) relatively poor S/B ratios are expected because they do not have strict on–off switches, and thus even basal physiological activities in cells can gradually elevate the background signals; (ii) a long stimulation time is necessary for the accumulation of the reporter, which limits monitoring of the temporal dynamics of molecular events of interest; and (iii) the detectable molecular events are limited because only events activating the reporter expression machinery can develop signals in the nucleus.

The second most popular and conceptually unique probe for imaging PPIs is bioluminescence resonance energy transfer (BRET). BRET is based on the nonradiative energy transfer from a luciferase (the energy donor) to a fluorescent protein (the energy acceptor) [7,8]. However, fluorescent proteins of BRET probes can exhibit one or multiple defects, as follows: (i) poor resonance energy transfer efficiency due to nonspecific aggregation of the fluorescent proteins [9]; (ii) slow maturation time of the fluorescent protein compared to the bioluminescent reporter, resulting in poor energy transfer efficiency; (iii) insufficient spectral separation and the corresponding cross-talks in the BL signals; and (iv) complexity in the molecular design of the probes because of the many factors influencing optical performance, including the dipole orientation, the valid distance for BRET within 100 Å, and the suitable overlap of the spectra of the two BRET partners [10].

A protein-fragment complementation assay (PCA) using split-luciferase is also a unique strategy for assaying PPIs in biological systems [11]. A luciferase in the PCA probes is dissected into N- and C-terminal fragments to measure the temporal loss of enzymatic activity. The fragments are fused to a pair of proteins of interest. When the two proteins of interest are approximated by PPIs, the adjacent luciferase fragments complement and recover the original enzymatic activity [12]. A split-NanoLuc-based PCA system named NanoBit is an important accomplishment in the study of PCAs and proved that the system is optimal for the accurate measurement of PPIs in cells [13]. However, its successful application in vivo is compromised by blue-wavelength light emission, which is poorly tissue-permeable. The artificially generated PCA fragments of the reporter luciferase generally recover merely 0.5–5% of the original enzyme activity via PPIs [14,15]. This drawback results in poor signal outputs. Another hurdle is that this strategy requires a sophisticated probe design and tedious optimization steps for deciding linker lengths and an optimal dissection site in the reporter luciferase sequence [11].

The overall defects discussed above were partially addressed by Kim et al., who developed molecular tension probes [16,17]. These probes were developed to index the molecular tension of a luciferase appended by intramolecular PPIs. The probe design is very simple and consists of a full-length luciferase sandwiched between two proteins of interest via minimal flexible linkers. If a ligand is eligible to trigger an intramolecular PPI between the proteins of interest, the PPI appends molecular tension to the sandwiched full-length luciferase and can enhance its enzymatic activities, i.e., the BL intensities. These tension probes were successfully applied to determine estrogen-activated PPIs between the estrogen receptor’s α ligand-binding domain (ER LBD) and the Src homology 2 (SH2) domain as its interacting partner [16]. Similarly, this strategy was also adopted to study rapamycin-mediated interactions between the FK506-binding protein (FKBP) and the FKBP rapamycin-binding protein (FRB) [17]. The imaging system developed using tension probes worked well even in a BRET system [8]. These molecular tension probes are unique but still have several limitations that need to be addressed. For example, the probes have limited versatility. They are conceptually able to only determine anti-parallel PPIs because two proteins inside the probe can anti-parallelly interact. Another problem is that the background BL intensities are relatively high because the probe contains a full-length luciferase that emits basal BL signals. This feature is a reason to worsen the S/B ratios.

In this study, we demonstrate a new genre of bioluminescent sensors for imaging PPIs, which is conceptually distinctive from any of the above-mentioned molecular imaging probes; thus, they are named molecular association assay (MAA) probes. The probe’s design is very plain, consisting of a full-length marine luciferase simply fused to a protein of interest with a flexible linker of minimal length. The interface region of this simple probe was simulated with ColabFold (AlphaFold-based) as an artificial intelligence (AI) tool for determining the optimal length of the flexible linker and/or the N-terminal region of the luciferase [18,19]. We found that this simple MAA probe alone surprisingly manages both roles of recognizing a ligand and quantitatively developing the BL intensities in mammalian cells. Our computational analysis of the binding modes suggests that the hinge region of the MAA probe is flexible before ligand binding but becomes stiff after ligand binding and protein association. We characterized the sensorial properties of representative MAA probes, FRB-ALuc16 or FRB-R86SG, with respect to the quantitative features, BL spectrum, and applicability for animal imaging. We also prove that the unique concept of an MAA can be applicable to studying other PPIs, such as the interactions of an ALuc16- or R86SG-fused ER LBDs with the SH2 domain. This strategy is conceptually distinctive from conventional ones such as PCA and BRET. Because we believe that the present MMA probes establish a new category of molecular imaging probes, we suggest that this work provides a new imaging repertoire for determining PPIs.

## 2. Materials and Methods

### 2.1. Reagents and Animals

The native coelenterazine (nCTZ) was custom-synthesized at our laboratory. From our previous studies, we obtained the cDNAs of the following proteins encoded in a pcDNA3.1(+) vector, i.e., *Renilla* luciferase 8.6-535SG (R86SG), artificial luciferase 23 (ALuc23 or A23; GenBank MF817968), artificial luciferase 49 (ALuc49 or A49; GenBank MF817976), and the fluorescent protein mPlum (Protein Data Bank (PDB) 2QLG_A) [8,20]. The cDNAs encoding the human FKBP (GenBank AAP36774.1), the human FRB (PDB 1AUE_A), the human ERα LBD (ER LBD, 305–550 aa), and the SH2 domain of *v*-Src (150–248 aa) were custom-synthesized by Eurofins Genomics (Tokyo, Japan) on the basis of sequence information from the public database NCBI [17].

NOD scid gamma (NSG) female mice (6 weeks old, ca. 20 g) were obtained from Charles River (Wilmington, MA, USA). The Administrative Panel on Laboratory Animal Care of Stanford University approved all procedures using laboratory animals in this study, and all experiments were conducted in accordance with the Guidelines for the Care and Use of Laboratory Animals (APLAC-26748, Approval Date: 11 September 2023).

### 2.2. Construction of the cDNA Constructs Encoding Various MAA Probes

**Figure 1:**
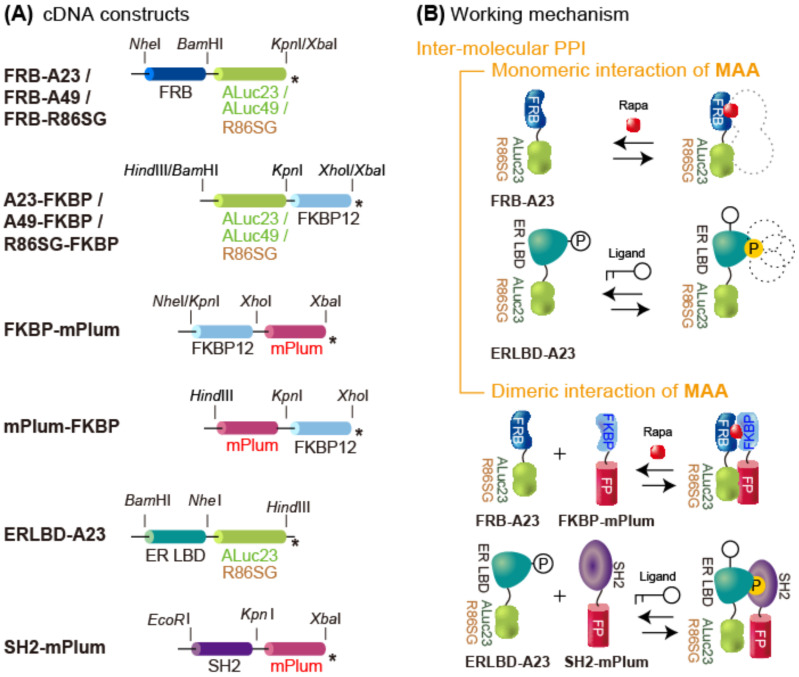
(**A**) Schematic illustration of the cDNA constructs of the molecular association assay (MAA) probes used in this study. The constructs are aligned in a way to highlight the characteristic domains in the probes. The probe names represent the protein domains from the N- to C-terminus, in order. The asterisks depict the stop codon. (**B**) Schematic illustration of the putative working mechanisms of representative MAAs in response to ligand stimulation. Abbreviations: FRB, FKBP-rapamycin-binding domain; FKBP, FK506-binding protein; ALuc23, artificial luciferase 23; ALuc49, artificial luciferase 49; R86SG, *Renilla* luciferase 8.6-535SG; Rapa, rapamycin; ER LBD, estrogen receptor ligand-binding domain; SH2, Src homology 2.

A series of cDNA constructs encoding MAA probes were generated through a tandem linkage of the cDNA blocks encoding the FRB, the FKBP, and marine luciferases (ALuc23, ALuc49, and *Renilla* luciferase 8.6-535SG (R86SG)), as shown in Figure 1A. For the probe’s design, we chose the R86SG variant of *Renilla* luciferase because it is the most advanced version of *Renilla* luciferase and a common marine luciferase with a strong optical signal. Similarly, we chose ALuc23 and ALuc49 as the artificial luciferase series as they are small and bright variants of copepod luciferases. In addition, we used flexible linkers with glycine and serine neutral amino acids, called a “GS linker”, to minimize steric hindrance between the domains and maximize the development of the ligand-activated signal.

Specifically, the cDNA blocks were generated using PCR and corresponding primers to introduce unique restriction sites, namely *Hin*dIII/*Bam*HI (to the FRB or the ER LBD), *Bam*HI/*Kpn*I (to the luciferases or mNep), or *Kpn*I/*Xho*I (to the FKBP, mPlum, or SH2), at the 5′ and 3′ ends, respectively. The cDNA blocks were cut by corresponding restriction enzymes and ligated in order into the mammalian cell expression vector pcDNA3.1(+). The fidelity of the cDNA constructs was confirmed with a genetic sequence analyzer by order (Eurofins Genomics). The MAA probes after expression were named FRB-A23, FRB-A49, FRB-R86SG (full length), A23-FKBP, A49-FKBP, R86SG-FKBP, FKBP-mPlum, mPlum-FKBP, ERLBD-A23, ERLBD-R86SG, and SH2-mNep, respectively.

The cDNA constructs encoding FRB-R86SG probe variants (*v*1–*v*4) were made using the following steps. First of all, cDNA fragments encoding R86SG (full length), R86SG (initial 1–8 AAs deleted), R86SG (initial 1–13 AAs deleted), and R86SG (9 GS linkers added and the initial 1–13 AAs deleted) were generated through PCR, using corresponding primers to introduce the unique restriction sites *BamH*I/*Kpn*I. The cDNA fragment encoding ALuc23 in the premade pcDNA3.1(+) vector was replaced with the one above encoding one of the R86SG variants. cDNA constructs were developed and encoded in the pcDNA3.1(+) vector, as illustrated in Figure 1A. The fidelity of the cDNA constructs was confirmed with a genetic sequence analyzer by order (Eurofins Genomics). The MAA probes after expression were named FRB-R86SG *v*1–*v*4, respectively.

### 2.3. Determination of the Fold Intensities of Various Combinations of MAA Probes

**Figure 2:**
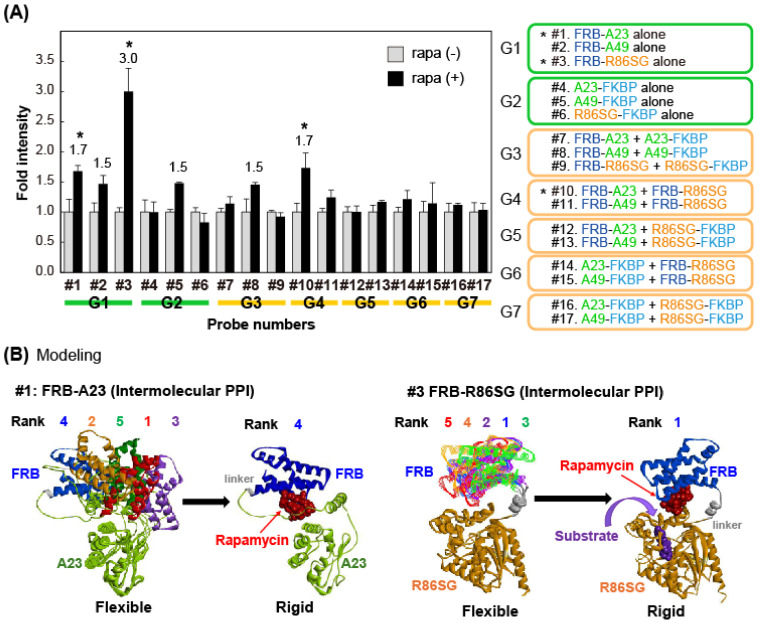
(**A**) Fold intensities of various combinations of MAA probes before and after the stimulation of rapamycin. The MAA probes are categorized into 7 groups according to the combinations. The asterisk marks highlight significant BL signals. The right panel specifies the detailed combinations. (**B**) Modeling of the binding modes of FRB-A23 (#1) and FRB-R86SG (#3) with rapamycin. The A23 and R86SG portions are shown in green and orange, respectively, while the movable FRB portions (ranks 1–5) are shown in various colors.

The fold intensities of various combinations of MAA probes were determined according to the following procedure (Figure 2A). COS-7 cells derived from African green monkey kidney fibroblasts were grown in 96-well microplates. The animal cells in the wells were transiently transfected with a pcDNA 3.1(+) plasmid encoding one of the MAA probes using a lipofection reagent (TransIT-LT1, Mirus Bio LLC (Madison, WI, USA)). The cells were then incubated overnight in a 5% (*v*/*v*) CO_2_ incubator (MG-71C, TAITEC co., Saitama, Japan). The wells containing the cells were conceptionally divided into two sections: one was stimulated with a vehicle solution (i.e., 0.1% (*v*/*v*) ethanol dissolved in a fresh culture medium), whereas the other was simultaneously stimulated with rapamycin dissolved in a fresh culture medium for 6 h (final concentration: 5 × 10^−7^ M). The vehicle was prepared by adding 0.1% (*v*/*v*) ethanol as the final concentration. Because the rapamycin stock solution was originally prepared with 100% ethanol, further dilution using a fresh culture medium resulted in an ethanol content of 0.1% (*v*/*v*) at the final concentration. Hence, to match the ethanol concentration in the solvent control, we used 0.1% ethanol in the medium.

The culture media in both sections were completely eliminated, and the remaining cells in both sections were lysed with a lysis buffer (Promega Co., Madison, WI, USA) for 20 min according to the manufacturer’s manual. The wells in both sections were then injected with PBS buffer containing nCTZ. The corresponding BL images were determined using the IVIS Spectrum imaging system and analyzed with the specific software Living Image version 4.7 (PerkinElmer, Waltham, MA, USA).

### 2.4. Determination of the Optical Properties of Combined MAA Probes in Lysates

**Figure 3:**
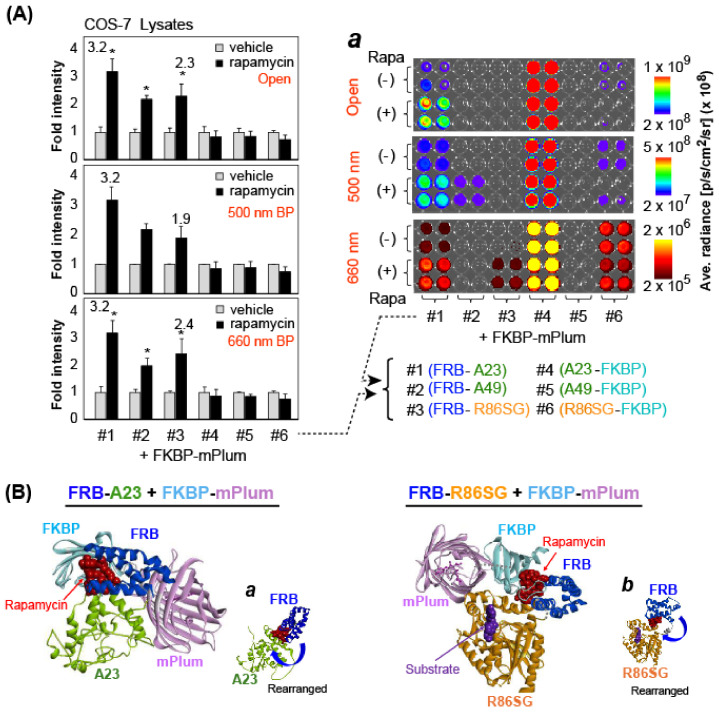
(**A**) Fold intensities of selected MAA probes after stimulation by rapamycin. The BL intensities of MAA probes determined with varying optical filters are shown. The asterisk marks highlight significant BL signals. The right panel (Inset ***a***) shows the corresponding optical images. (**B**) Putative binding modes of [FRB-A23 + FKBP-mPlum] and [FRB-R86SG + FKBP-mPlum] pairs. Rearrangements from single MAAs are highlighted in Insets ***a*** and ***b***, respectively.

Inspired by the results in Figure 2A, we further investigated the optical properties of combined MAA probes (i.e., FKBP-mPlum plus one of the MAA probes in groups 1 and 2) in lysates according to the following procedure (Figure 3). COS-7 cells were prepared using the same protocol as in Figure 2A. The animal cells in 96-well microplates were transiently cotransfected with pcDNA3.1(+) plasmids encoding FKBP-mPlum and one of the MAA probes in groups 1 and 2, as shown in the X-axis of Figure 3A. The cells were placed overnight in a 5% (*v*/*v*) CO_2_ incubator. The microplate wells culturing the cells were conceptionally categorized into two sections. The first section was stimulated with a vehicle (0.1% (*v*/*v*) ethanol dissolved in a fresh culture medium) and the second section was activated by rapamycin dissolved in a fresh culture medium for 6 h (final concentration: 5 × 10^−7^ M). The culture media were completely decanted, and the well-attached cells were lysed with the lysis buffer (Promega) for 20 min according to the manufacturer’s instructions. The wells were then injected with PBS buffer containing nCTZ. The corresponding BL images were determined using the IVIS Spectrum imaging system with filters (open, 500 nm bandpass (BP) filter, and 660 nm BP filter) and analyzed with Living Image version 4.7.

### 2.5. Structural Modeling of MAA Probes and Their Binding with Rapamycin

The structures of the FRB-A23 and FRB-R86SG probes were modeled here using ColabFold (AlphaFold-based) [18]. For each of the five models, the rapamycin-bound structure (PDB 1FAP) [21] was superimposed onto the FRB portion to create the structure of the binding mode. Then, for the complexes with FKBP-mPlum, the four domains (FRB-A23 or -R86SG, and FKBP-mPlum) of the two fused probes were manually positioned (by deleting the linker) so that the FRB and FKBP sandwiched the rapamycin. In addition, to investigate the difference in binding between FKBP-mPlum and mPlum-FKBP to FRB-R86SG, mPlum was repositioned to have the N-to-C order of mPlum-FKBP.

For the N-terminal regions of R86SG fused to the FRB of the four variants (*v*1–*v*4), two crystal structures of *Renilla* luciferase were obtained from the protein data bank (PDB 2PSJ [22] and 7OMR [23] and the AlphaFold structure (AF-P27652-F1-model_*v*4) from the AlphaFold database (https://alphafold.ebi.ac.uk/ (16 August 2024)). Then, they were superimposed. All the structures in this study were visualized using BIOVIA Discovery Studio 2017 R2 (Dassault Systèmes, Vélizy-Villacoublay, France).

### 2.6. Determination of the BL Spectra of Selected MAA Probes in Pairs

**Figure 4:**
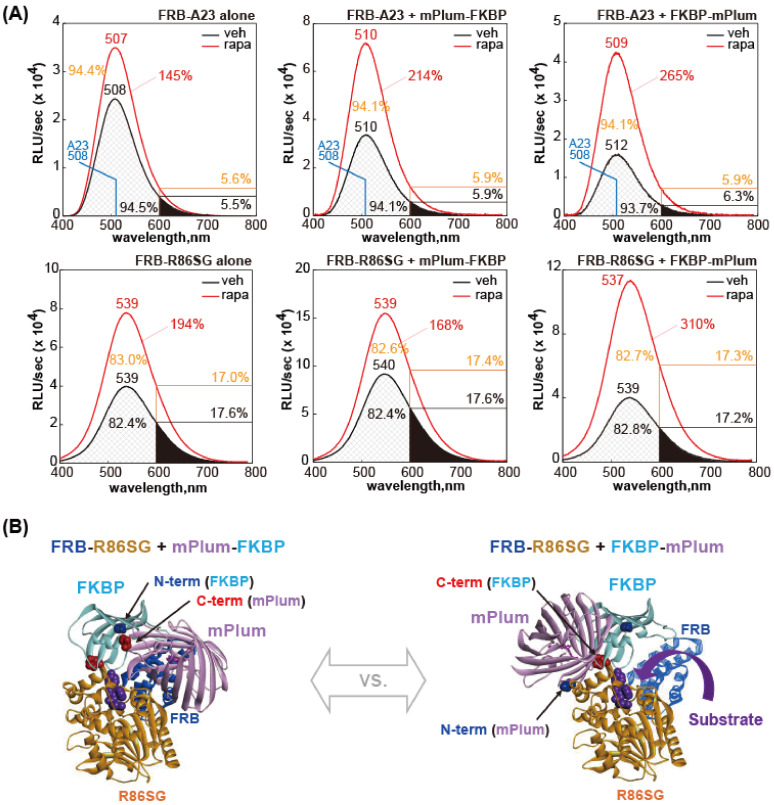
(**A**) The BL spectral changes of various association probes in response to rapamycin. A spectral area longer than 600 nm is depicted by vertical lines. The percentages indicate the portions of the spectral area shorter or longer than 600 nm. Numbers on the peaks show the λ_max_ values. (**B**) Binding mode of the [FRB-R86SG and mPlum-FKBP] pair, compared to that of the [FRB-R86SG and FKBP-mPlum] pair.

The BL spectra of selected MAA probes were investigated according to the following method (Figure 4). COS-7 cells were grown in 6-well microplates and transiently transfected with pcDNA3.1(+) vector(s) encoding the following MAA probes: (i) FRB-A23 alone, (ii) FRB-A23 and mPlum-FKBP, (iii) FRB-A23 and FKBP-mPlum, (iv) FRB-R86SG alone, (v) FRB-R86SG and mPlum-FKBP, and (vi) FRB-R86SG and FKBP-mPlum.

The cells were incubated in a 5% (*v*/*v*) CO_2_ incubator for 1 day and subcultured into 12-well microplates. The cells were then conceptionally divided into two sections. One section was stimulated overnight with a vehicle (0.1% (*v*/*v*) ethanol dissolved in a fresh culture medium) or rapamycin dissolved in a fresh culture medium (final concentration: 5 × 10^−7^ M). The culture media were completely eliminated and the remaining cells on the well surface were lysed with a lysis buffer (Promega) for 20 min according to the manufacturer’s instructions. Forty μL of each cell lysate was aliquoted into a 200 μL PCR tube. After injection of 40 μL of the substrate nCTZ solution dissolved in PBS, the PCR tube was mounted on a precision spectrophotometer (AB-1850, ATTO, Tokyo, Japan), and the corresponding BL spectra were immediately determined. The spectra were analyzed with Excel (Microsoft, Redmond, WA, USA) and Igor Pro 7 (WaveMetrics, Portland, OR, USA).

### 2.7. Determination of the Optical Properties of Combined MAA Probes in Live Cells

**Figure 5:**
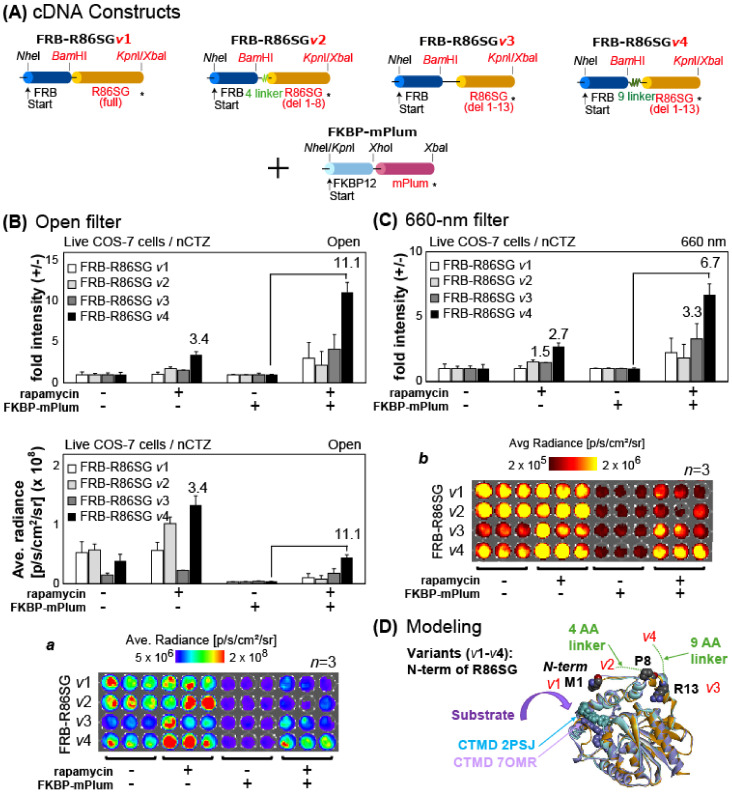
(**A**) Schematic illustration of the cDNA constructs encoding various MAA probes. The asterisks depict the stop codon. (**B**) Fold intensities of FRB-R86SG *v*1–*v*4 in the presence or absence of rapamycin and/or FKBP-mPlum. The bottom panel (Inset ***a***) shows the corresponding BL images obtained with the open filter. (**C**) Fold intensities of FRB-R86SG *v*1–*v*4 in the presence or absence of rapamycin and/or FKBP-mPlum. The bottom panel (Inset ***a***) shows the corresponding BL images obtained with the 600 nm BP filter. (**D**) R86SG *v*1–*v*4, highlighting the N-terminal regions. The three N-terminal amino acids (M1, P8, and R13) connecting to the linkers of *v*1, *v*2, and *v*3/*v*4, respectively, are highlighted in spheres, and the coelenteramide (CTMD) in the crystal structures are represented by spheres.

We were inspired by the promising results of the combination of [FRB-R86SG and FKBP-mPlum] and tried to optimize the optical properties by pairing various versions of FRB-R86SG probes with their counterpart FKBP-mPlum in live COS-7 cells (Figure 5).

Plain COS-7 cells were prepared using the same protocol as in Figure 2A and conceptually split into two parts. The first part comprised cells in a 96-well microplate that were transiently introduced with the pcDNA3.1(+) vector encoding one of the MAA probes carrying R86SG variants (i.e., FRB-R86SG *v*1–*v*4). The other cells were transiently cotransfected with pcDNA3.1(+) vectors encoding FKBP-mPlum and one of the MAA probes containing R86SG variants (i.e., FRB-R86SG *v*1–*v*4). The cells were kept overnight in a 5% (*v*/*v*) CO_2_ incubator. The wells maintaining the cells were conceptionally grouped into two sections. The sections were stimulated with a vehicle (0.1% (*v*/*v*) ethanol dissolved in a fresh culture medium) or rapamycin dissolved in a fresh culture medium for 6 h (final concentration: 5 × 10^−7^ M). The culture media were completely decanted, and the cells in the vacant wells were then immersed in PBS buffer containing the substrate nCTZ. The corresponding BL images were immediately determined using the IVIS Spectrum imaging system equipped with filters (open and 660 nm BP filter) and analyzed with Living Image version 4.7.

### 2.8. Cell-Based Imaging of the Molecular Associations Between the ER LBD and the SH2 Domain

**Figure 6:**
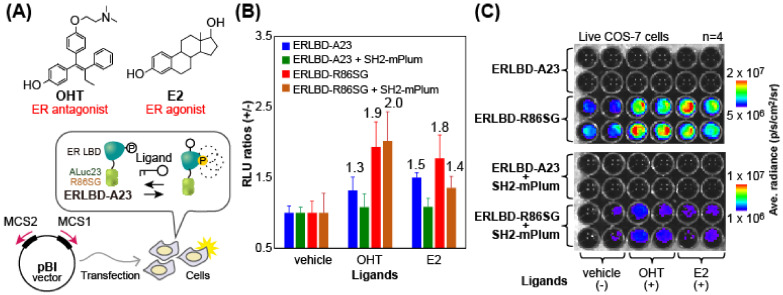
Determination of the estrogenicity of ligands with ER LBD-based MAA probes. (**A**) Putative molecular imaging mechanism of ER LBD-based MAA probes and the chemical structures of an estrogen agonist and an antagonist. (**B**) Relative BL intensities of the MAA probes in response to specific ligands. (**C**) The corresponding BL images of the MAA probes on a 96-well black-frame microplate.

The PPI between the ER LBD and the SH2 domain was determined with four kinds of specially designed MAA probes according to the following procedure (Figure 6): COS-7 cells cultured in a 96-well black-frame microplate were transiently transfected with the pBI CMV1 vector (Takara Bio, San Jose, CA, USA) encoding one of the following MAA probes, i.e., (i) ERLBD-A23 alone, (ii) ERLBD-R86SG alone, (iii) a pair consisting of [ERLBD-A23 and SH2-mNep], and (iv) a pair consisting of [ERLBD-R86SG and SH2-mNep]. The cells were then incubated in a CO_2_ incubator (Sanyo, Tokyo, Japan) until the MAA probes were overexpressed in the cells. The cell media were then completely eliminated, and the remaining cells were immersed for 60 min with an aliquot (50 μL) of fresh culture media, dissolving one of the following ligands, i.e., the vehicle (0.1% DMSO), 17β-estradiol (E_2_), or 4-hydroxytamoxifen (OHT) (final concentration of each ligand: 1 μM). The culture media were carefully eliminated from the microplate and the remained cells were simultaneously injected with fresh media, dissolving the substrate (nCTZ) using a 12-channel micropipette (Gilson, Madison, WI, USA). The corresponding optical intensities from the live cells were immediately determined using the IVIS Spectrum imaging system with Living Image ver. 4.7.2.

### 2.9. Determination of the BL Intensities of Mice Carrying an MAA Probe

**Figure 7:**
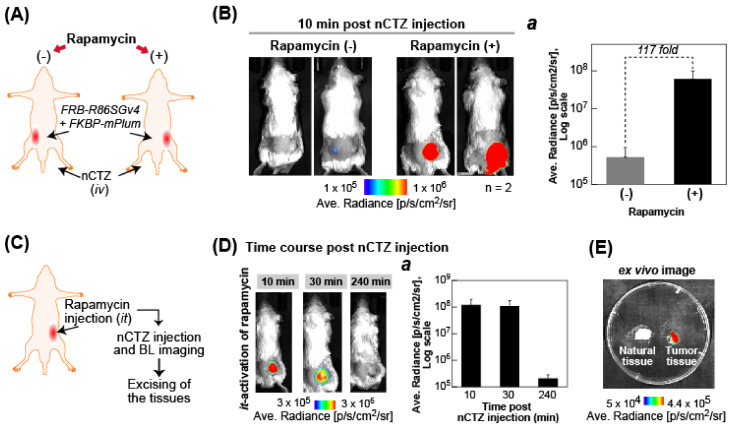
(**A**) Schematic illustration of the in vivo experimental protocol. (**B**) In vivo bioluminescence imaging (BLI) of the tumor cells carrying FRB-F86SG *v*4 and FKBP-mPlum without and with rapamycin activation. The right panel (Inset ***a***) shows the corresponding BL intensities from the tumors implanted in mice (*n* = 2). (**C**) Schematic representation of the protocol of the in vivo time-course experiment. (**D**) Time course of the BL intensities of a representative mouse bearing the tumor carrying FRB-F86SG *v*4 and FKBP-mPlum after the intratumoral (*it)* administration of rapamycin. The right panel (Inset ***a***) shows the ROI measurement of the BL intensity from the mouse tumors (*n* = 2). (**E**) Ex vivo BL image of the tumor carrying FRB-F86SG *v*4 and FKBP-mPlum or normal tissues excised from mice after *it* injections of rapamycin and the *iv* administration of nCTZ.

The rapamycin-activated BL intensities were determined with NOD scid gamma (NSG) mice as a breed of immunodeficient laboratory mice carrying an MAA probe (Figure 7). In our study, we used 4 mice in total, with 2 in each of the groups (*n* = 2) for the probe signal measurements in vivo. This is based on our previous studies, where we have observed minimum variability in most probe imaging studies.

All animal handling was performed in accordance with Stanford University Institutional Animal Care and Use Committee guidelines (APLAC-26748, Approval Date: 11 September 2023) and by adherence to the NIH Guide for the Care and Use of Laboratory Animals.

We cultured 10 × 10^6^ of plain COS-7 cells initially in a 6-well plate and transiently transfected them with pcDNA 3.1(+) vectors encoding FRB-F86SG *v*4 and FKBP-mPlum. The cells were then incubated overnight in a 5% (*v*/*v*) CO_2_ incubator (Thermo Fisher Scientific, Waltham, MA, USA). The cells were then harvested by trypsinization and centrifugation, and an equal amount of the cells were subcutaneously (*sc*) implanted into the left or right flanks of mice. After housing the mice overnight in a cage to stabilize the cells, the mice were randomly divided into two groups. One group was intratumorally (*it*) injected with the vehicle (1% ethanol dissolved in PBS), and the other group was *it* injected with rapamycin (10 mg/kg) (*n* = 2). The mice were then rested in a cage overnight, and further intravenously (*iv*) injected with nCTZ (10 mg/kg). The corresponding BL images were obtained using the Lago X imaging system (Spectral Instruments Imaging, Tucson, AZ, USA) with a 60 s exposure time.

The time course of the BL intensities from mice carrying the same rapamycin-stimulated tumor was also determined (Figure 7C–E). The mouse was prepared using the same method as in Figure 7A,B. After the mouse was *iv* injected with nCTZ (10 mg/kg), the corresponding BL images were then monitored at 10, 30, and 240 min. The ex vivo BL images were further determined from the tumor and normal tissue excised from the mouse and placed in a Petri dish.

## 3. Results and Discussion

### 3.1. Motivation to Investigate a New Genre of Simple, Potentially Bioluminescent Probes

We previously reported a basic concept of “molecular strain probes” [17,24] and their modified BRET probes [8]. However, this approach has a critical limitation that is not generally applicable to PPIs, i.e., molecular strain probes conceptually determine only anti-parallel PPIs. In addition, we previously witnessed a small clue that the negative control FRB-A23 alone may develop a weak BRET-free signal [8]; however, nobody was aware of the importance back then (Figure S1).

The background described above motivated us to intensively investigate a new genre of potentially bioluminescent probes that are simpler and generally applicable for PPI imaging in this study. We developed various cDNA constructs, as illustrated in Figure 1A, and the potential working mechanisms after expression, as shown in Figure 1B, are discussed in detail in the following sections.

### 3.2. The Molecular Association of Proteins Can Be Indexed with BL in Mammalian Cells

We first categorized the MAA probes under seven groups according to their molecular designs and combinations, namely G1–G7 (Figure 2A). G1 and G2 contain a single MAA probe, whereas G3–G7 were designed to contain two MAA probes. The results showed that the probes in G1 (#1–#3) surprisingly enhanced the BL intensities upon stimulation by rapamycin. The highest fold intensity of ca. 3.0 was obtained by FRB-R86SG (#3), followed by FRB-A23 (#1) with a fold intensity of 1.7. A49-FKBP (#5) was the only probe that showed elevated BL intensities in G2. Among the pairs in G3–G7, the combination of FRB-A23 and FRB-R86SG (#10) significantly elevated the BL intensity in response to rapamycin and the fold intensity was approximately 1.7. The combination of FRB-A49 and A49-FKBP (#8) caused a weak elevation in the BL intensity. In contrast, the other combinations did not exert significant increases in the BL intensity in response to rapamycin.

The overall results are summarized as follows. (i) A full-length luciferase can be sensitive enough to elevate enzymatic activities in response to adjacent molecular associations in mammalian cells, (ii) the optical performance of G1, when compared to G2, reveals that the simplest MAA probe should be a fusion protein in the format of an FRB followed by a full-length luciferase, and (iii) the relatively poor optical performance of G3 and G5–G7 shows that FKBP-fused luciferases are not optimal designs for sensing rapamycin-triggered associations with the proteins of interest.

Rapamycin-activated FRB–FKBP binding is a well-known model PPI, but this does not clearly explain why only some of the MAA probes in Figure 2 are activatable by rapamycin. To elucidate the reason and the optimal linker length, we computationally investigated the binding modes of FRB-A23 (#1) and FRB-R86SG (#3) with rapamycin (Figure 2B). When the structures of these probes (FRB-A23 and FRB-R86SG) were predicted using ColabFold (AlphaFold-based) [18], it was found that the FRB and luciferase in both probes had flexible conformations with the linkers. Among these models, the fourth-ranked model of FRB-A23 was optimal for the rigid and steric hindrance-free binding of rapamycin with FRB-A23. Similarly, the top-ranked model of FRB-R86SG was optimal for rapamycin binding without steric hindrance. These results suggest that the probe, which has a flexible conformation in the apo-state, is stabilized by rapamycin binding by forming a rigid structure, as shown in a previous study [24]. The authors speculate that (i) this ligand-driven rigidity of FRB-A23 and FRB-R86SG and (ii) the optimal linker length for minimizing the steric hindrance and for accommodating the ligand collectively work as an “on–off switch” for the probe system. The highest S/B ratios of FRB-R86SG (Figure 2A) can be explained with the structural modeling shown in Figure 2B, i.e., the rigid conformation facilitates the substrate’s access into the active site of R86SG, whereas the flexible conformation constitutes active site-blocking modes.

Besides the ligand-driven rigidity, we suggest that the ligand-bound MAA probes can recruit endogenous FKBP in cells, which may additionally contribute to the probe’s rigidity. This view is supported by references that endogenous FKBP is a ubiquitous and abundant protein in mammalian cells [25]. Thus, it has the potential to bind the MAA probes. In addition, no other association forms are supposed because ligand-activated FRB-A23 or FRB-R86SG does not make a homodimer, although FKBP does [26,27].

### 3.3. Combining FKBP-mPlum with FRB-A23 or FRB-R86SG Exerts Better Optical Performance as 2MAA Systems

As the quantitative determination of PPIs in mammalian cells is of great impact, we investigated the best counterpart of the successful FRB-A23 and FRB-R86SG (Figure 3). Because FKBP-fused luciferases (G3 and G5–G7) showed poor optical performance compared to FRB-fused luciferase in Figure 2A, we alternatively tested FKBP-mPlum as the counterpart of FRB-fused luciferase (full length). The results in Figure 3A showed that FRB-fused marine luciferases (#1–#3) exert better fold intensities with FKBP-mPlum, whereas FKBP-fused marine luciferases (#4–#6) did not initiate a considerable elevation of BL intensities upon rapamycin stimulation.

The corresponding BL images in Inset ***a*** of Figure 3A show that the highest BL intensities were obtained with the combination of A23-FKBP (#4) and FKBP-mPlum, but their fold intensities upon induction with rapamycin (+/−) were significantly low. In contrast, the combination of FRB-A23 (#1) and FKBP-mPlum developed both considerable absolute and high fold BL intensities (ca. 3.2). The combination of FRB-R86SG (#3) and FKBP-mPlum was relatively poor regarding absolute BL intensities but showed a significantly enhanced fold intensity (ca. 1.9–2.4) in response to rapamycin. Notably, the intensity change was clearly observed with a 660 nm BP filter. These results show that the combinations of FRB-A23 (#1) or FRB-R86SG (#3) with FKBP-mPlum enhance the BL signals in response to rapamycin, and combining an MAA probe with an interacting counterpart makes an excellent molecular imaging tool for determining PPIs. When FKBP-mPlum is selected as the potential BRET-emitting counterpart of FRB-A23 or FRB-R86SG, the bandpass filtering indicates that the BRET signals are not significant upon rapamycin stimulation. This poor BRET signal suggests that the overexpressed FKBP could be increasing the photon output of the luciferase regardless of the mPlum fluorescent protein used in the construct.

The intermolecular interactions of FKBP-mPlum with FRB-A23 and FRB-R86SG were structurally investigated to determine the putative binding modes (Figure 3B). Considering that rapamycin is sandwiched between the FRB and FKBP, each of which is made flexible owing to the linkers, the mPlum portion should be repositioned next to each luciferase (A23 or R86SG). The rearrangements of FRB-A23 and FRB-R86SG are indicated in Insets *a* and *b* of Figure 3B, respectively. In each probe combination, we found that (i) the optical signal is maximized when the overall association is stable, and (ii) the association works in a way to facilitate the substrate’s access to the active sites of the luciferase (A23 or R86SG). This explains why the BL intensities are enhanced.

### 3.4. The BL Spectra of the MAA Probes Are Differentially Enhanced by Rapamycin

We further investigated the BL spectra of MAA probes in various experimental conditions (Figure 4). The BL spectra of FRB-A23 (#1) alone or FRB-R86SG (#3) alone confirmed that the BL intensities were commonly enhanced in a rapamycin-dependent manner, and the spectral peak (color) was not altered by the rapamycin stimulation (Figure 4A, left column). Both FRB-A23 and FRB-R86SG commonly showed significantly decreased background intensities in the presence of FKBP-mPlum. Therefore, the overall S/B ratios were improved upon rapamycin induction (Figure 4A, right column). However, these background-lowering effects of the spectra were commonly not significant when combined with mPlum-FKBP (Figure 4A, middle column).

The BL spectral peaks (λ_max_) of FRB-A23 were observed to range from 507 to 512 nm, while those of FRB-R86SG were between 537 and 540 nm. The λ_max_ values of mPlum-FKBP and FKBP-mPlum were not influenced significantly by the addition of rapamycin. Although no obvious BRET signals from the combinations were observed in the spectra, the right shoulders (on the side with longer wavelengths) commonly had a tailing intensity feature. Because FRB-A23 generated green light, the spectral portion longer than 600 nm (P_600_) was approximately 5.6–5.9%. In contrast, FRB-R86SG emits green light with a longer wavelength; thus, the P_600_ ranges from 17.0 to 17.4%.

To understand the dramatic reduction in the background of FRB-R86SG with FKBP-mPlum, the binding modes of the pairs were structurally investigated, i.e., the [FRB-R86SG and mPlum-FKBP] pair and the [FRB-R86SG and FKBP-mPlum] pair (Figure 4B). When FKBP-mPlum and mPlum-FKBP were compared, it was found that FKBP-mPlum facilitated better substrate entry, whereas the mPlum in mPlum-FKBP was positioned closer to the substrate entrance of R86SG, suggesting that it sterically hinders the access of the substrate to R86SG. In addition, with regard to A23, *Gaussia princeps* luciferase (GLuc), a homolog of A23, was previously shown to have highly flexible conformations using NMR analysis [28], which hampered the association of the FRB-A23 and mPlum-FKBP pair (without background lowering) here. However, we speculate that the positioning of mPlum in the pair also influences the substrate’s entry.

### 3.5. Combination of FKBP-mPlum with FRB-R86SG Variants Exerts the Highest Fold Intensities in Live Cells

As FRB-R86SG (#3) enhanced the BL intensities with considerable fold intensity in the above experiments, we further investigated an optimized version of [FKBP-mPlum and FRB-R86SG] pairs for imaging PPIs (Figure 5). The results show that FRB-R86SG *v*1–3 alone showed a slightly elevated BL intensity when exposed to rapamycin, and FRB-R86SG *v*4 alone, in particular, showed the highest fold intensity of ca. 3.4 in live cells. Furthermore, FRB-R86SG *v*1–*v*4 paired with FKBP-mPlum enhanced the overall fold intensities in live cells, and the highest fold intensity of ca. 11.1 was surprisingly obtained by pairing FRB-R86SG *v*4 with FKBP-mPlum. The corresponding BL images revealed that supplemented FKBP-mPlum dramatically decreases the background BL intensities, thus eventually maximizing the fold intensities in live mammalian cells. Upon comparison of the absolute intensities, the [FRB-R86SG *v*4 and FKBP-mPlum] pair emitted the highest BL intensities compared to the other pairs.

Inspired by the ligand-activated BL intensities of the FRB-R86SG *v*1–*v*4, we computationally modeled the molecular structures of the N-terminal regions of R86SG in the probes (Figure 5D). In the two crystal structures of *Renilla* luciferase and the AlphaFold model structure, the N-termini of R86SG stretch out toward their substrate entrance. The linker modeling revealed that the full-length variant of R86SG (*v*1) and the four-amino-acid linker variants of R86SG (*v*2) obviously hinder the substrate entry. In addition, the 13-AA deleted variant of R86SG (*v*3) was directly fused to the FRB, and this short FRB–R86SG linkage caused lower responsiveness to the ligand rapamycin. In fact, the average radiance was significantly low in variant *v*3 (Figure 5B). On the other hand, the variant of R86SG (*v*4), carrying a long nine-amino-acid (GS) linker, has increased flexibility to recruit rapamycin without steric hindrance and with better substrate accessibility compared to the R86SG (*v*3) variant. These analyses explain why the variant of R86SG (*v*4) exerts a higher fold intensity than the others.

In this study, we designed the probes FRB-R86SG *v*1–*v*4, inspired by our previous success stories in which the N-terminally deleted R86SGs were effective for improving the S/B ratios, such as the eight-amino-acid deleted one [20] and the 13-AA deleted one [24], at the N-terminal end of R86SG. The authors understand that the shortened N-terminal end of R86SG and the long flexible GS linker collectively contributed to the improvement of the S/B ratios.

### 3.6. Determination of the General Applicability of the Basic Concept of MAAs with a Modified Version of MAA Probes Containing the ER LBD

As the basic concept of MAA probes successfully worked with FRB-fused marine luciferases, we additionally determined if the concept is generally applicable to the other PPI models, e.g., interactions between the ER LBD and the SH2 domain (Figure 6). We chose this model PPI between the ER LBD and the SH2 domain because it is an important molecular event called the “non-genomic pathway” of the ER, where the agonist or antagonist can induce phosphorylation at site Y537 of helix 12 of the ER LBD, which is recognized by the adjacent SH2 domain [29].

The results showed that ERLBD-R86SG alone enhances the BL intensities by up to 1.9- and 1.8-fold under the stimulation of OHT and E2, respectively, whereas the same cells do not elevate the BL intensities under the stimulation of the vehicle (0.1% (*v*/*v*) DMSO solution). Likewise, ERLBD-A23 showed the same ligand-driven elevation of BL intensities, but the overall absolute intensities were approximately 10-fold weaker than those of ERLBD-R86SG. In parallel, PPIs between overexpressed MAA probes and SH2-mPlum were determined using the same protocol as the one above, where the fusion proteins were equivalently expressed because the cDNA constructs were encoded in the bidirectional expression vector, pBI CMV1. The results show that the pair of [ERLBD-R86SG and SH2-mPlum] surely elevated the BL intensities by up to 2.0-fold in response to OHT. However, the overall absolute intensities were approximately 10-fold weaker than those of ERLBD-R86SG alone. The pair of [ERLBD-A23 and SH2-mPlum] weakly drifted the BL intensities under the stimulation of OHT and E2, but the signal elevation was not significant.

The overall results indicate that (i) the basic concept of an MAA probe is generally applicable to the other association models, such as the binding between the ER LBD and the SH2 domain, (ii) simple tagging of a full-length luciferase to a protein of interest can make the simplest molecular imaging probe, here named an MAA, and (iii) this strikingly simple design of an MAA greatly relieves the burdens of researchers regarding the time and labor involved in the probe development process.

It is challenging to decipher the reason why ER LBD-fused R86SG alone works as an optical probe for estrogens. The authors suggest seeking the reason from the innate signal transduction pathways of the ER. It is well-known that ligand-activated ER makes a dimer, recruits many coactivators, and is translocated into the nucleus for gene expression (the genomic pathway). In parallel, the ER provokes various kinases to be activated in the cytosol, which is so rapid that they cannot be related to direct gene expression (the nongenomic pathway) [30,31]. The authors see that all these intrinsic molecular associations, which lead to a stiff structure after the ligand binding of the ER LBD, can work as an on–off switch in the simplest optical probe. The elevated BL intensity naturally reflects the estrogenicity of the ligands.

### 3.7. Rapamycin-Driven PPIs Were Determined in Living Mice Carrying an MAA Probe

Because the [FRB-R86SG *v4* and FKBP-mPlum] pair showed the highest S/B ratio in the previous section, we further investigated the PPIs of this pair in an animal model (Figure 7).

The BL images show that rapamycin-activated mice emit obviously brighter BL signals than when the vehicle alone is administered. The specific BL intensities from the right frank LOIs were approximately 117-fold stronger (Figure 7) than the left frank LOIs of the mice. This result is interpreted as rapamycin diffusing to the tumor cells while carrying the [FRB-R86SG *v4* and FKBP-mPlum] pair and triggering the association of FRB-R86SG *v4* and FKBP-mPlum in the tumors of the mice. This rapamycin-activated association finally developed BL intensities stronger than those stimulated by the vehicle.

The time-course results in Figure 7C,D reveal that the BL intensities developed with rapamycin are sustainable for approximately 30 min and return to their basal intensities 240 min post-administration. The ex vivo study in Figure 7E confirms that the BL images were generated from the tumor carrying the [FRB-R86SG *v4* and FKBP-mPlum] pair, not from the normal tissues.

The present animal experiments used only four mice for each measurement (*n* = 2), which can be considered an insufficient sample size for arriving at statistical significance in the conclusion. However, in most sensor imaging studies, we have observed minimum variability between animal imaging results. Hence, we used a few animals to validate the utility of our probe system for in vivo imaging applications in this study.

## 4. Conclusions

To date, many conventional optical probes have been developed for assaying molecular events in physiological systems. However, they require sophisticated probe designs and tedious optimization steps, such as (i) deciding optimal linker lengths and dissection sites in the reporter luciferase or fluorescent protein and (ii) determining the optimal layout of the probe components for the best sensor performance.

To tackle these common drawbacks, the present study provides a breakthrough using the simplest molecular imaging probe design, which is just a full-length marine luciferase simply fused to a protein of interest with a flexible linker. The present study demonstrated that this simplest probe alone surprisingly works by recognizing a specific ligand and emitting the BL signal in both mammalian cells and a mouse model. We characterized the sensor properties of representative MAA probes, FRB-ALuc23 and FRB-R86SG, in detail in vivo and in vitro. We demonstrated that the concept of an MAA can be applicable to other PPI models, such as ALuc16- or R86SG-fused ER LBD models that emit BL signals in response to estrogenicity.

We applied an AlphaFold-based AI tool to simulate the binding modes and working mechanisms, as well as decide the optimal linker length and N-terminal end. This AI-based analysis suggests that (i) the hinge region of the MAA probe is flexible before ligand binding but becomes stiff after ligand binding and protein association and that (ii) the association of MAA probes with other proteins facilitates the substrate’s access to the active sites of the luciferase (ALuc23 or R86SG).

This molecular imaging strategy is conceptually distinctive from conventional ones such as PCA and BRET. Considering the conceptual uniqueness and versatility of this approach, we are expecting widespread applications for these probes as a new imaging repertoire to determine PPIs in living organisms.

## Data Availability

Data are contained within the article and Supplementary Materials.

## Supplementary Materials

The following supporting information can be downloaded at: https://www.mdpi.com/article/10.3390/bios15050299/s1, Figure S1: (A) The optical intensity variance of the various fragmented BL probes according to ligands in living mammalian cells (left: open filter, right: Cy5.5 filter). This negative control result was reproduced from Ref. [8] with permission from the Royal Society of Chemistry (RSC). In the negative control study, four different probes were examined if ligand can elevate BL intensities: i.e., FRB-A23 is deficient of FKBP, whereas FKBP-A23 or FKBP-mPlum is lacking FRB. FRB-A23 weakly elevates the BL intensity. In contrast, FRB-A23-FKBP (named TP2.4) carrying both FRB and FKBP exerts the strongest BL intensities in response to rapamycin. (B) Illustration of the working mechanisms of the three different BL probes. (C) The chemical structures of ligands used in this study.
