# Supplementary material for: Molecular Association Assay Systems for Imaging Protein–Protein Interactions in Mammalian Cells
Source: Biosensors (Basel). 2025 May 8;15(5):299. doi: 10.3390/bios15050299 (PMC12109908; doi:10.3390/bios15050299)
Supplement: Supplementary file 1 [file biosensors-15-00299-s001.zip › biosensors-3499864-supplementary.pdf]

Suppl. Information

Molecular Association Assay Systems for Imaging Protein–Protein Interactions in Mammalian Cells

Sung-Bae Kim [1,*], Tadaomi Furuta [2], Suresh Thangudu [3], Arutselvan Natarajan [3], Ramasamy Paulmurugan [3]

[1] Environmental Management Research Institute, National Institute of Advanced Industrial Science and Technology (AIST), 16-1 Onogawa, Tsukuba 305-8569, Japan; kimu-sb@aist.go.jp
[2] School of Life Science and Technology, Institute of Science Tokyo, B-62 4259 Nagatsuta-cho, Midori-ku, Yokohama 226-8501, Japan; furuta@bio.titech.ac.jp
[3] Molecular Imaging Program at Stanford, Bio-X Program, Stanford University School of Medicine, Palo Alto, California 94304, USA; suresh07@stanford.edu (S.T.); anataraj@stanford.edu (A.N.); paulmur8@stanford.edu (R.P.)

* Correspondence: kimu-sb@aist.go.jp

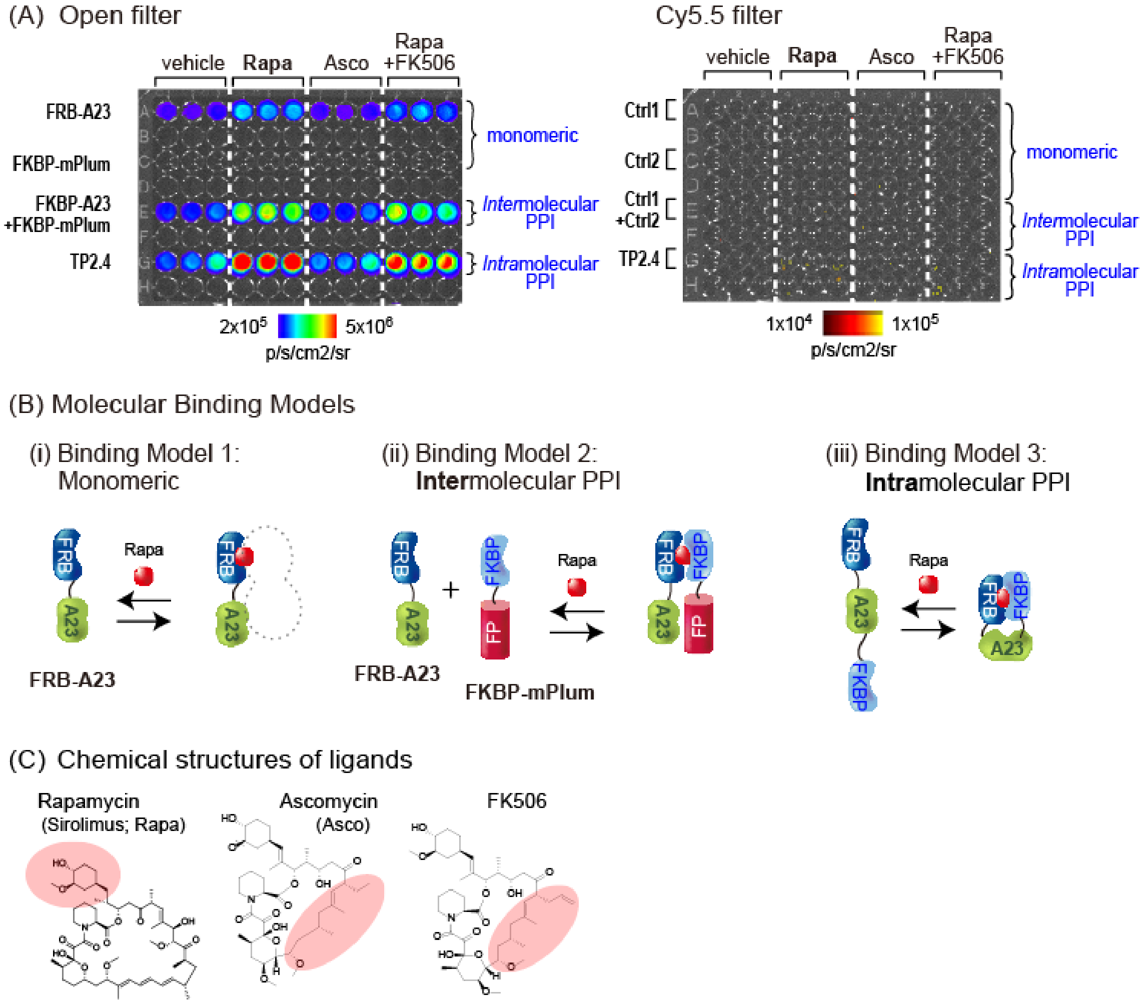

Suppl. Figure 1. (A) The optical intensity variance of the various fragmented BL probes according to ligands in living mammalian cells (left: open filter, right: Cy5.5 filter). This negative control result was reproduced from Ref. 8 with permission from the Royal Society of Chemistry (RSC). In the negative control study, four different probes were examined if ligand can elevate BL intensities: i.e., FRB-A23 is deficient of FKBP, whereas FKBP-A23 or FKBP-mPlum is lacking FRB. FRB-A23 weakly elevates the BL intensity. In contrast, FRB-A23-FKBP (named TP2.4) carrying both FRB and FKBP exerts the strongest BL intensities in response to rapamycin. **(B)** Illustration of the working mechanisms of the three different BL probes. **(C)** The chemical structures of ligands used in this study.
